# Lateral approach for insertional Achilles tendinitis with Haglund deformity

**DOI:** 10.3389/fsurg.2022.1063833

**Published:** 2023-01-06

**Authors:** Jiantao Jiang, Cheng Wang, Shaoling Fu, Jiazheng Wang, Chenglin Wu, Guangxiao Yao, Guoxun Song, Wenqi Gu, Kai Yang, Jianfeng Xue, Zhongmin Shi

**Affiliations:** ^1^ Department of Orthopaedic Surgery, Shaoxing Shangyu Traditional Chinese Medicine Hospital, Zhejiang, China; ^2^ Department of Orthopaedic Surgery, Shanghai Sixth People's Hospital, Shanghai, China

**Keywords:** Achilles tendinitis, Haglund deformity, lateral approach, surgery, detachment

## Abstract

**Objective:**

The study aims to investigate the functional outcome of the lateral approach for insertional Achilles tendinitis (IAT) with Haglund deformity.

**Methods:**

From January 2016 to September 2019, 14 cases of IAT with Haglund deformity that resisted conservative treatment received surgery in our department. A lateral approach was used to debride the bony and soft tissue and reattach the insertion of the Achilles tendon. The Visual Analog Scale (VAS), American Orthopedic Foot and Ankle Score (AOFAS), and Victorian Institute of Sport Tendon Study Group-Achilles Tendinopathy score (VISA-A) were used to evaluate clinical outcomes.

**Result:**

The mean patient age was 39.57 years at the time of surgery. The mean follow-up was 14.74 months. The mean VAS score significantly decreased from 4.86 ± 0.86 preoperatively to 1.21 ± 1.58 postoperatively (*P* < 0.001). The mean AOFAS score significantly improved from 66.64 ± 6.23 preoperatively to 90.21 ± 11.50 postoperatively (*P* < 0.001). The mean preoperative and the last follow-up VISA-A were 66 (range 56.75–69.25) and 86 (range 75.75–97.00) points, respectively (*P* < 0.05).

**Conclusion:**

The lateral approach was effective and safe for IAT with Haglund deformity. Moreover, the mid-term functional outcome was promising.

**Level of Clinical Evidence:**

IV

## Introduction

Insertional Achilles tendinitis (IAT) was first described in 1992 by Clain and Baxter (1). The inflammation and the degeneration that appeared within the Achilles insertion are the hallmarks of IAT. Haglund deformity was an abnormally prominent posterosuperior calcaneal deformity first described in 1928 (2). Theoretically, the association between the IAT and Haglund's deformity exist, because the deformity may irritate the Achilles and retrocalcaneal bursa. Historically, many researchers have tried to figure out the association by measuring calcaneal shape, such as the Fowler–Phillip angle (3), Bohler's angle, and Chauveaux–Leit angle (4), but to our knowledge, no literature supports the effect of calcaneal shape on IAT symptoms.

The non-operative treatment for IAT involves shoe modification, non-steroidal anti-inflammatories, activity restriction, and physical therapy (5), but as reported, the failure rate for non-operative methods was as high as 50%–60% (6). Most operative procedures include removal of pathologic tendon and calcifications, the posterosuperior calcaneal prominence, and the retrocalcaneal bursa (7). There are different approaches: the longitudinal midline approach, the lateral approach, the Cincinnati approach, and the minimal endoscopic approach. Many literature works have proven the effectiveness of the approach mentioned above (8–11). To our knowledge, the central longitude splitting approach was most likely to be used, but the complications, such as scar irritation, limit its application.

The aim of this retrospective study was to analyze the effectiveness of the lateral incision to treat IAT with Haglund deformity.

## Patients and methods

From January 2016 to September 2019, 14 cases of IAT with Haglund deformity that resisted to conservative treatment received surgery in our department. A lateral approach was used to debride the bony and soft tissue, and reattach the insertion of the Achilles tendon. All the patients in the study failed non-operative treatment and had a minimum of 6 months before surgery. The American Orthopedic Foot and Ankle Society-Hindfoot Scale (AOAS-HF) (12), Visual Analog Pain Scale (VAS) (13), and the Victorian Institute of Sport Assessment-Achilles questionnaire (VISA-A) (14) were used preoperatively and at final follow-up to evaluate the clinical outcome. AOFAS is the main scale of clinical efficacy evaluation, including three aspects of pain, function, and force line. A VAS score was used to evaluate the degree of pain before surgery and at the last follow-up. The VISA-A questionnaire is reliable for comparing patients with varying degrees of severity of Achilles tendinopathy, with results ranging from 0 to 100. It asks a total of eight questions in the areas of pain, daily functioning, and physical activity. Inclusion criteria and exclusion criteria were listed below. We had access to information that could identify individual participants during or after data collection.

### Ethical considerations

The retrospective study was registered at Clinical Trials Registry (approval no. ChiCTR1900020941) and approved by the Ethics Committee of Shanghai Sixth People's Hospital. This study was performed in accordance with the principles of the Declaration of Helsinki. The written consent approval was obtained from all enrolled participants, and their privacy rights were respected. Also, the manuscript is in accordance with the recommendations for the conduct, reporting, editing, and publication of scholarly work in medical journals.

### Inclusion criteria

(1) Symptomatic history ≥6 months.
(2) Age ≥ 18 years.
(3) Non-operative treatment is ineffective.
(4) Accompanied by Haglund deformity.
(5) A follow-up duration ≥ 6 months.

### Exclusion criteria

(1) Have not been properly treated by non-operative methods [types of non-operative treatment: resting or braking, reducing the amount of exercise appropriately, using cold compresses, non-steroidal anti-inflammatory agents (NSAIDs), hormone injection therapy, using orthopedic shoes or foot pads, physiotherapy, and Achilles tendon pulling training]. Duration of non-operative treatment: a minimum of 6 months before surgery.
(2) IAT without Haglund deformity.
(3) Patients with diabetes, and autoimmune diseases (such as ankylosing spondylitis, rheumatoid arthritis, and gout).
(4) Patients have a history of Achilles trauma.
(5) Patients who only have medial heel pain.

### Surgery tips

**Figure 1:**
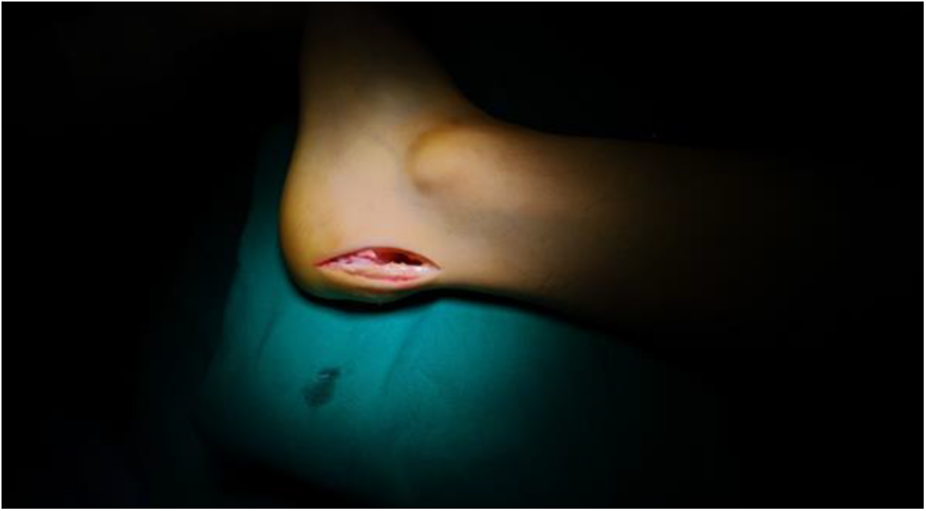
The lateral incision approach: 5 mm anterior to the lateral border of the Achilles tendon and 2 cm proximal to the superior crest of the calcaneus and extended to the insertion of the Achilles tendon.

**Figure 2:**
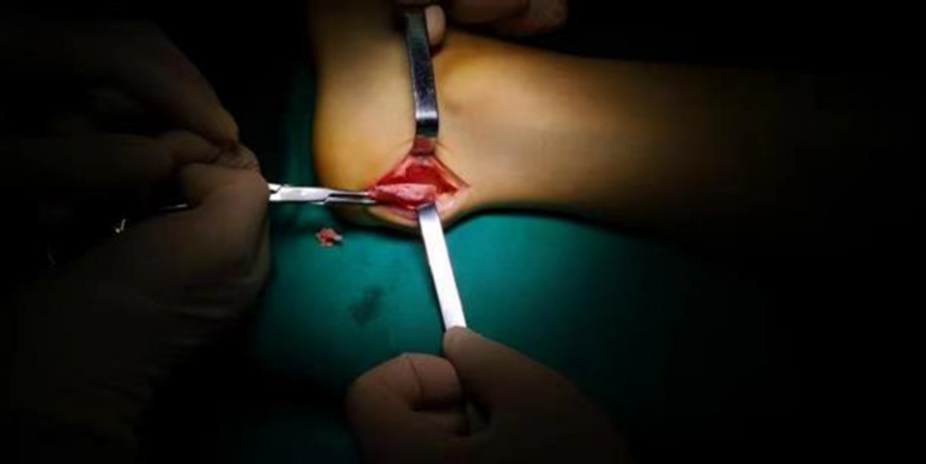
Calcaneal exostectomy after Achilles insertion detachment and debridement.

**Figure 3:**
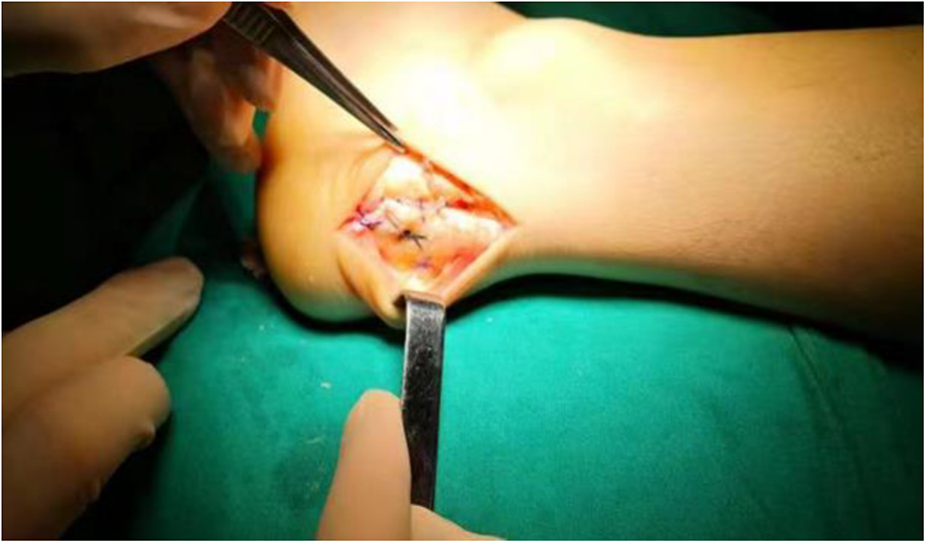
Achilles insertion reattachment using a single-row suture anchor technique.

The lateral approach was located 5 mm anterior to the lateral border of the Achilles tendon, and the incision started 2 cm proximal to the superior crest of the calcaneus and extended distally to the insertion of the Achilles tendon on the calcaneus (Figure 1. The lateral incision approach: 5 mm anterior to the lateral border of the Achilles tendon and 2 cm proximal to the superior crest of the calcaneus and extended to the insertion of the Achilles tendon). All surgeries were performed by the same senior foot and ankle surgeon. The initial dissection was taken directly down to the bone, with full-thickness flaps and sub-periosteal detachment of the entire Achilles insertion. The pathological retrocalcaneal bursae was excised. A calcaneal exostectomy was performed to remove the Haglund deformity, followed by debridement of the insertion of the Achilles tendon (Figure 2). Due to the fact that the Haglund deformity was excised under direct vision, intraoperative fluoroscopy was not used in the surgery. The Achilles tendon was then reattached to the calcaneus using a single-row suture anchor technique (Figure 3. Achilles insertion reattachment using a single-row suture anchor technique).

### Postoperative care

**Figure 4:**
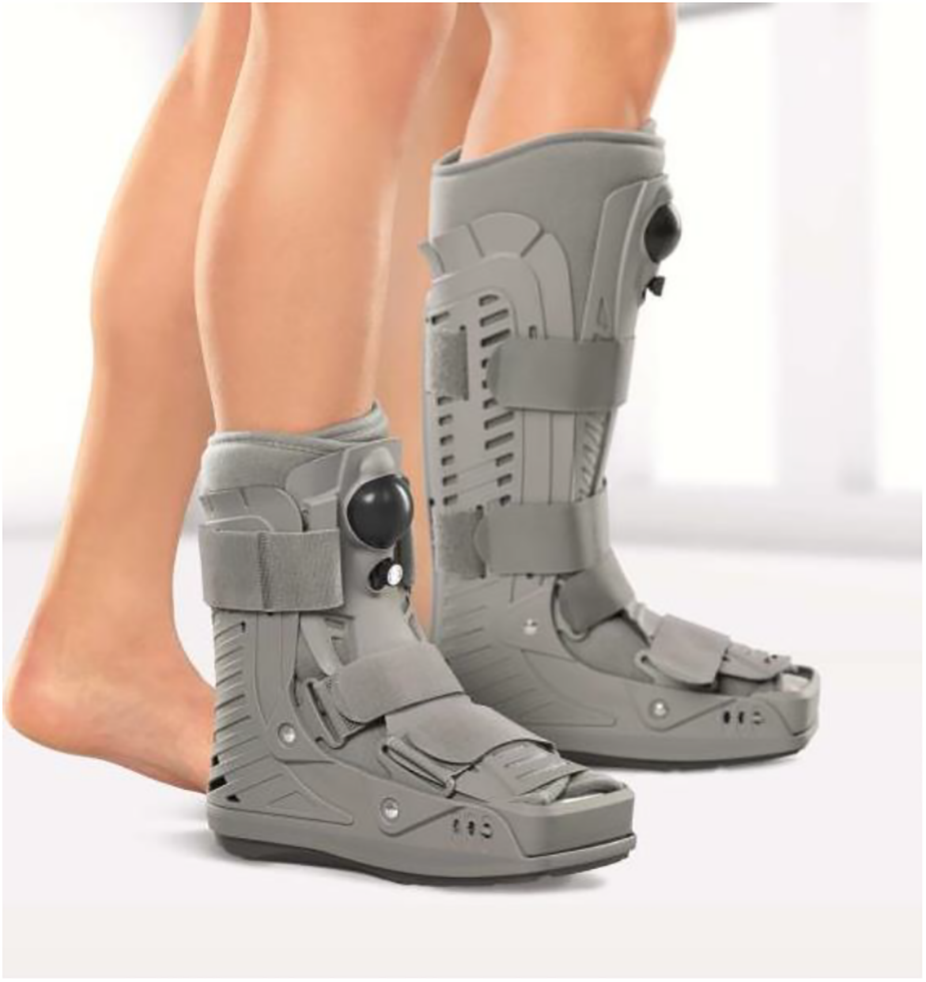
Patients were suggested to wear a postoperative walking boot.

The postoperative follow-up examinations were performed by the surgeon. All patients used bulged dressings for 2 weeks before stitches were removed. During this period, they were instructed to be non-weight-bearing. After 2 weeks, the patients were suggested to wear a postoperative walking boot (Figure 4. It provides stability while slightly guiding and supporting, which is beneficial to postoperative recovery) and the patients started weight-bearing partially and rehabilitation exercise. After 6 weeks, patients could tolerate 100% weight-bearing and wear their own shoes. They were not allowed to participate in strenuous activities for 3 months after surgery.

### Statistical analysis

The Statistical Package for Social Sciences, version 25.0 for Windows (SPSS, Chicago, IL, USA), was used for statistical analysis. The mean VAS and AOFAS scores and standard deviations of each group were calculated and compared with each other using the paired-samples *T*-test. The median VISA-A scores were calculated and compared using the Mann–Whitney *U*-test.

## Results

We collected the data on patients diagnosed with IAT from January 2016 to September 2019. JTJ and SF, who were two of the researchers, served as outcome assessors and no conflict of interest was reported. According to the inclusion and exclusion criteria, 14 patients (14 feet, 8 left, and 6 right) were included in this study, and all of them were followed up. The wounds all healed in one stage, without infection. The mean patient age was 39.57 (range 18–61) with females (14.29%) and males (85.71%). The nine of them had a history of smoking. The 14 patients had a mean BMI of 26.19, with a range of 19.53–31.38. Postoperatively, the patients were followed up for a mean of 14.74 (range 6–30) months.

The VAS scores preoperatively and at final follow-up were 4.86 ± 0.86 (range 4–6) points and 1.21 ± 1.58 (range 0–5) points, respectively (*P* < 0.001). The mean AOFAS-HF score at preoperative and final follow-up were 66.64 ± 6.23 (range 55–77) points and 90.21 ± 11.50 (range 61–100) points, respectively. The VISA-A questionnaire was used to evaluate the clinical severity of Achilles tendinopathy. The mean VISA-A scores preoperatively and at final follow-up were 66 (range 56.75–69.25) and 86 (range 75.75–97.00) points, respectively (*P* < 0.001) (Table 1). No patients suffer from incisional inflammation or Achilles tendon rupture postoperatively.

**Table 1:** Different scale scores preoperative and at the last follow-up.

Mean preoperative and postoperative VAS, AOFAS, and VISA scores			
	VAS	AOFAS	VISA-A
Preoperative	4.86±0.86	66.64±6.23	66 (56.75–69.25)
The last follow-up	1.21±1.58	90.21±11.50	86 (75.75–97.00)
*t*/*z*	9.787	−6.368	−3.109
*P*-value	<0.0001	<0.0001	0.002

Note: VISA-A does not conform to normal distribution; VAS and AOFAS were in accordance with normal distribution.

One patient reported that she had moderate discomfort with shoe wear. Through physical examination, we found a keloid at the distal site of the incision. We suppose that the suture irritation of the soft tissue and scar hyperplasia result in discomfort. Two patients suffered from moderate pain every day, and seven patients reported mild pain occasionally. The two patients reported that the pain was nearly as severe as preoperative, but the characteristics were not the same, and surprisingly, all the seven patients referred to the “occasion” as rainy and snowy days.

## Discussion

The most important finding in the research was the promising outcome of the surgery using a lateral incision. The reason we chose the lateral incision was the convenience of exposing the whole Haglund deformity. We could easily get a certain size of Haglund deformity and excise it under direct vision. Some researchers worry about the sural nerve injury (15), but according to a Cadaveric study (16), the approach we take is very safe. Compared with the central splitting approach (17), the approach we take is likely to cause less scar irritation. Recently, a study reported the complications following the midline incision approach for IAT, 41% of patients had problems finding the right shoe, 32% reported a shoe conflict, and these shoe-related problems were predominantly due to scar pain (60%) (18). The one in our study complained about the discomfort with shoe wear because of the distal incision keloid (diameters of about 1 cm). Two weeks after we excised the keloid and the knot remained under the skin, the patient felt good when wearing shoes. Alternatively, a transverse Cincinnati incision has the advantages of adequate exposure, shorter incision, and less scar irritation (8). A novel technique of minimally invasive calcaneal osteotomy (19) was used in patients who had insertional Achilles tendinopathy associated with Haglund deformity and got a promising outcome compared with open Haglund resection. Based on the study, the patients just received osteotomy without Achilles debridement and reconstruction, so the approach may not be useful when the Achilles degeneration is severe. Several studies (5, 20, 21) have proven the effectiveness of endoscopic approach. Compared with other approaches, it appears to have less risk of scar irritation, wound infection, and sural nerve injury; however, in our practice, it is difficult to excise the Haglund deformity and debride the tendon calcification under an endoscope, and we could not excise the introachilles lesion.

**Figure 5:**
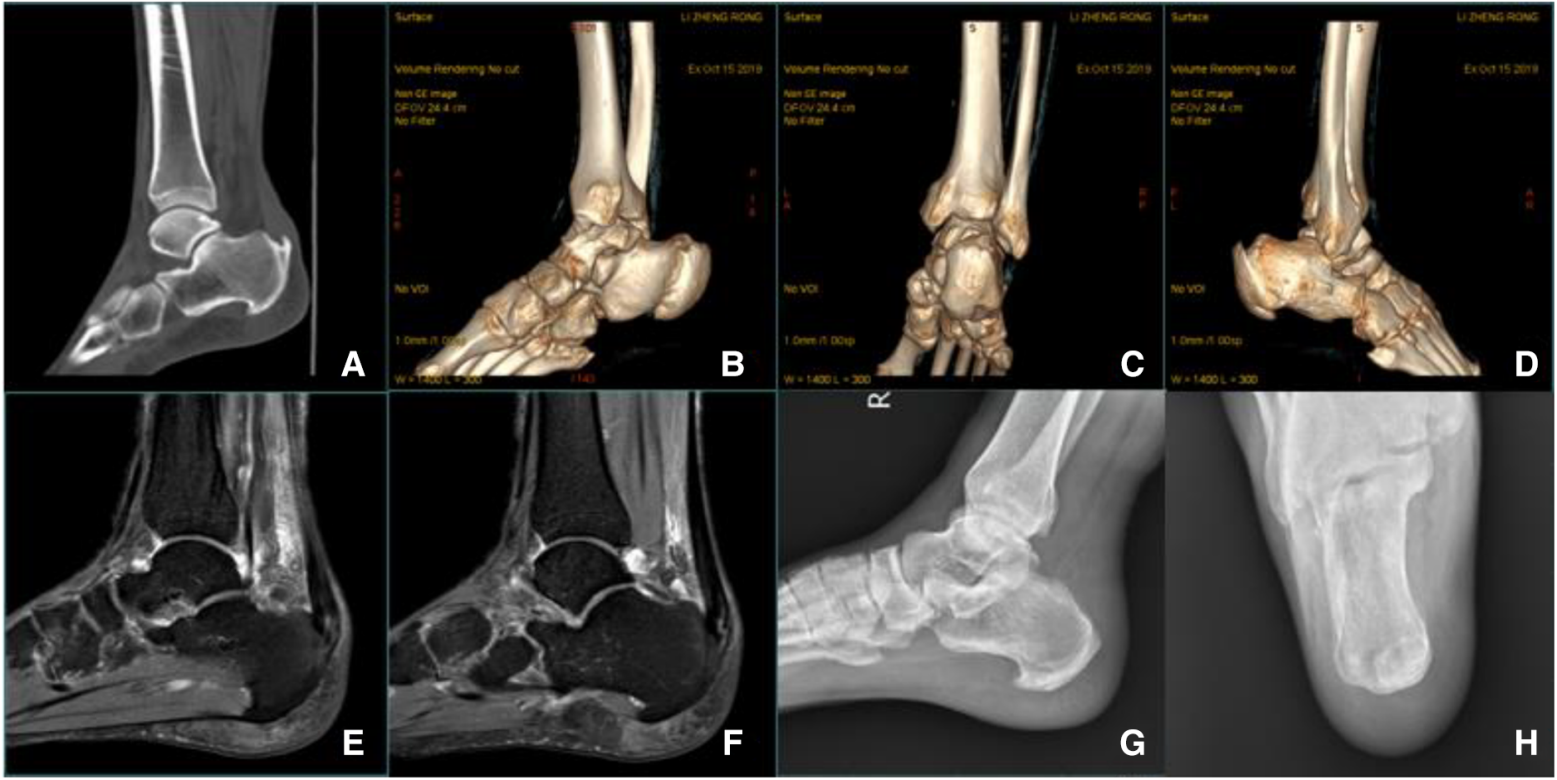
(**A–D**) Preoperative CT scan: Calcification of Achilles tendon insertion was evident, accompanied by Haglund deformity. (**E, F**) Preoperative MR imaging: Denaturation and calcification at the insertion of the Achilles tendon, fluid accumulation behind the ankle joint, and compression of the flexor hallucis longus tendon. (**G, H**) Postoperative radiography: The posterior calcaneus was flat, and there was no Haglund deformity at the insertion of the Achilles tendon.

In our study, the Haglund deformity was completely excised under direct vision followed by smoothing of the resultant osteotomized surface (Figure 5. Preoperative and postoperative imaging data). We proposed that the abnormal friction and impact caused by Haglund deformity in daily activities accelerate the Achilles' degeneration, and some researchers agreed with the opinion that repeated pressure from ill-fitting footwear or the deformity itself can cause retrocalcaneal bursitis (22). Different osteotomies were introduced in patients who have heel pain associated with Haglund deformity and reported the promising outcome (23–25), but they only processed osteotomies; the Achilles were not stripped from the calcaneus, debrided, and reconstructed. Additionally, for these osteotomies, flattening of the heel in a cavus foot (10) and loss of fixation (19) may be the risks. In Jun Young Choi's study (19), at the final follow-up at ≥18 months, some heel pain persisted, although radiographic union and deformity correction were successfully achieved. We suspected that the results were caused by the Achilles pathological changes.

The amount of acceptable detachment for Achilles insertion varies in different literature. Some reports said that up to 50% of detachment was safe (26), and for those detachment greater than 50%, double-row fixation was recommended (5, 27). In our study, through physical examination combined with radiography, we determined that lateral heel pain existed in all patients, and detachment and debridement were performed as needed. Through the lateral approach, we detach the Achilles insertion from the lateral side, and the medial Achilles lesion can be easily seen and treated after we detach the lateral Achilles insertion. The single-row suture anchor technique was used to make the Achilles reinsertion, and there was no Achilles tendon rupture and avulsion in our study.

There are only a few study about the function outcome of postoperative IAT using a lateral incision. Lin et al. (28) concluded that calcaneoplasty and reattachment of the Achilles tendon *via* a lateral approach for insertional tendinopathy enable early weight-bearing and achieve a good outcome and pain relief. Xia et al. (29) reported the lateral approach provided better short-term pain relief and reduced delayed wound healing compared with the central approach, while other outcomes were comparable. In our series of 14 patients who underwent tendon detachment, debridement, and reattachment for IAT and were followed up for a mean of 14 months, the mean AOFAS ankle–hindfoot score improved from 67 to 90, the mean VAS score declined from 4.82 to 1.21, and the VISA-A score improved from 62 to 82. These results were comparable with the above studies.

There are some limitations to the lateral incision we introduced. First, this incision is not appropriate for patients who only have medial heel pain. In other words, the degeneration only exists in the medial part of the Achilles. Second, when transposition of flexor hallucis longus was needed, this incision could not expose the flexor hallucis longus clearly. There are some limitations to the study. This is a retrospective study from a single institution, which may result in selection and observational biases. Additionally, the relatively small sample size and the short follow-up duration may influence the final result. More samples and longer follow-up duration may contribute to a more credible long-term outcome.

In conclusion, the lateral incision and the technique we introduced were effective for IAT associated with Haglund deformity, and the mid-term functional outcome was promising.

## Data Availability

The original contributions presented in the study are included in the article/Supplementary Material, further inquiries can be directed to the corresponding author/s.
